# Transcriptome Profiling Reveals Role of MicroRNAs and Their Targeted Genes during Adventitious Root Formation in Dark-Pretreated Micro-Shoot Cuttings of Tetraploid *Robinia pseudoacacia* L.

**DOI:** 10.3390/genes13030441

**Published:** 2022-02-27

**Authors:** Saleem Uddin, Muhammad Zeeshan Munir, Sadia Gull, Aamir Hamid Khan, Aimal Khan, Dilawar Khan, Muhammad Asif Khan, Yue Wu, Yuhan Sun, Yun Li

**Affiliations:** 1 National Engineering Laboratory for Tree Breeding, Key Laboratory of Genetics and Breeding in Forest Trees and Ornamental Plants of Ministry of Education, Beijing Advanced Innovation Center for Tree Breeding by Molecular Design (BAICFTBMD), Engineering Technology Research Center of Black Locust of National Forestry and Grassland Administration, College of Biological Sciences and Technology, Beijing Forestry University, Beijing 100083, China; saleemkhan86@hotmail.com (S.U.); wuyuea555@163.com (Y.W.); syh831008@163.com (Y.S.); 2 School of Environment and Energy, Shenzhen Graduate School, Peking University, Shenzhen 518055, China; jiger007@pku.edu.cn; 3 School of Horticulture and Plant Protection, Yangzhou University, Yangzhou 225009, China; sadiagull137@yahoo.com; 4 National Key Laboratory of Crop Genetic Improvement, Huazhong Agricultural University, Wuhan 430070, China; passaban786@outlook.com; 5 University of Chinese Academy of Sciences, Beijing 100049, China; aimal_khan_p@yahoo.com; 6 State Key Laboratory of Plant Genomics, Institute of Genetics and Developmental Biology, The Innovative Academy of Seed Design, Chinese Academy of Sciences, Beijing 100101, China; 7 School of Soil and Water Conservation, Beijing Forestry University, Beijing 100083, China; dilawarafridi333@hotmail.com; 8 Key Laboratory of Silviculture and Conservation, Beijing Forestry University, Beijing 100083, China; asifkhanbaluch@yahoo.com

**Keywords:** tetraploid *Robinia pseudoacacia* L., dark pretreatment, adventitious rooting, miRNA-seq, RNA-seq

## Abstract

Tetraploid *Robinia pseudoacacia* L. is a difficult-to-root species, and is vegetatively propagated through stem cuttings. Limited information is available regarding the adventitious root (AR) formation of dark-pretreated micro-shoot cuttings. Moreover, the role of specific miRNAs and their targeted genes during dark-pretreated AR formation under in vitro conditions has never been revealed. The dark pretreatment has successfully promoted and stimulated adventitious rooting signaling-related genes in tissue-cultured stem cuttings with the application of auxin (0.2 mg L^−1^ IBA). Histological analysis was performed for AR formation at 0, 12, 36, 48, and 72 h after excision (HAE) of the cuttings. The first histological events were observed at 36 HAE in the dark-pretreated cuttings; however, no cellular activities were observed in the control cuttings. In addition, the present study aimed to uncover the role of differentially expressed (DE) microRNAs (miRNAs) and their targeted genes during adventitious root formation using the lower portion (1–1.5 cm) of tetraploid *R. pseudoacacia* L. micro-shoot cuttings. The samples were analyzed using Illumina high-throughput sequencing technology for the identification of miRNAs at the mentioned time points. Seven DE miRNA libraries were constructed and sequenced. The DE number of 81, 162, 153, 154, 41, 9, and 77 miRNAs were upregulated, whereas 67, 98, 84, 116, 19, 16, and 93 miRNAs were downregulated in the following comparisons of the libraries: 0-vs-12, 0-vs-36, 0-vs-48, 0-vs-72, 12-vs-36, 36-vs-48, and 48-vs-72, respectively. Furthermore, we depicted an association between ten miRNAs (novel-m0778-3p, miR6135e.2-5p, miR477-3p, miR4416c-5p, miR946d, miR398b, miR389a-3p, novel m0068-5p, novel-m0650-3p, and novel-m0560-3p) and important target genes (auxin response factor-3, gretchen hagen-9, scarecrow-like-1, squamosa promoter-binding protein-like-12, small auxin upregulated RNA-70, binding protein-9, vacuolar invertase-1, starch synthase-3, sucrose synthase-3, probable starch synthase-3, cell wall invertase-4, and trehalose phosphatase synthase-5), all of which play a role in plant hormone signaling and starch and sucrose metabolism pathways. The quantitative polymerase chain reaction (qRT-PCR) was used to validate the relative expression of these miRNAs and their targeted genes. These results provide novel insights and a foundation for further studies to elucidate the molecular factors and processes controlling AR formation in woody plants.

## 1. Introduction

Tetraploid *Robinia pseudoacacia* L. (*R. pseudoacacia* L.) is a leguminous, deciduous, ornamental tree species that is artificially produced by doubling the chromosomes, with the application of colchicine, in diploid cells of the congeneric species, known as black locust (2n = 92). *R. pseudoacacia* L. is native to south Korea, and was introduced into China in 1997 [1,2]. Economically, this species has a significant role in supplying wood production, honey production, and feed for animals; it also favors the rapid fixation of elite genotypes [3,4]. Furthermore, tetraploid *R. pseudoacacia* L. is highly adaptable to harsh environments, including cold, drought, salt, pest infestation, and nutrient deficiencies [5], which increases its economic value for further research studies [6]. The propagation of these species is difficult due to its long generation periods, and thus life cycle, which is a limiting factor.

Currently, clonal propagation through stem cuttings is the only way to deploy genetically improved tetraploid *R. pseudoacacia* L. varieties [7]. Several reports have also shown that in vitro propagation of tetraploid *R. pseudoacacia* L. is an effective method to produce large numbers of clonal plants [8], because woody species are usually more difficult to root than herbaceous plants [9].

There are different types of roots, such as the primary root, secondary root, crown root, and adventitious roots (AR). Roots that are formed spontaneously, or from non-rooted tissues, are known as AR. AR formation is an inheritable quantitative trait in plants controlled by multiple endogenous and environmental factors, such as the genetic background of the maternal plant; the application of exogenous hormones; and environmental conditions, such as light and etiolation [10,11].

The partial or complete absence of light for a specific time period is termed etiolation [12]. Etiolation is known to significantly induce AR formation in different species, causing partial rejuvenation [11,13,14,15]. Etiolation changes the physiology, anatomy, and molecular mechanisms of different tree species [16]. Moreover, etiolation regulates essential hormone-related genes and hormone levels [17]. For example, increased Indole-3-acetic acid (IAA) levels and expression of indole-3-pyruvate monooxygenase YUCCA (YUC) and auxin efflux carrier (PIN) genes were observed in *Arabidopsis* seedlings [18]. CYP90 levels increased under weak light throughout brassinosteroid (BR) biosynthesis during petiole elongation in *Arabidopsis* [19]. Hormonal signaling induces many metabolic processes that depend on light signals during plant growth and development [20]. During the light signal transduction pathway, hormone action occurs in a downstream manner [21].

Additionally, plant hormones are also the most important modulators of AR formation [22]. Auxins play an essential role during AR development in many plants. Among auxins, indole-3-butyric acid (IBA) is the most widely employed exogenous auxin used for the stimulation of AR formation. However, the molecular mechanisms by which auxin regulates the process of AR formation are poorly understood. In cuttings of many plants, auxins (IBA) play a crucial role in inducing AR [23]; it is experimentally proven in *Arabidopsis* that IBA acts mainly via conversion to the biologically active IAA in the cuttings [24]. However, in some plant species, such as petunia [25], adventitious roots are produced without chemical stimulation, and are instead controlled by polar auxin transport and local accumulation of the endogenous auxin (IAA) in the rooting zones. The treatment of cuttings with the IBA has been reported to significantly improve the rooting rate in tetraploid *Robinia pseudoacacia* L. [12], *Juglans regia* L. [26], *Pinus contorta* [27,28], *Malus pumila* [29], and *Pinus radiata* [30]. Regarding in vitro induction of AR formation, IBA is considered to be more stable and effective than IAA, and is widely used in vegetative propagation [22]. Moreover, additional phytohormones, including ethylene, cytokinins, jasmonate, abscisic acid, and gibberellin, act in concert with auxin in a complex regulatory network of AR formation [31]. Overall, these observations have revealed the essential role of auxin in the complex process of AR formation.

Deep sequencing [32,33] has improved our understanding of these complex biological processes of AR formation at a different levels, especially at the genetic level. However, information at the level of miRNA and their targets is still limited; analyzing gene expression is an effective way to improve understanding of the development of AR at the molecular level.

MicroRNAs are universal, highly conserved, 20–24 nucleotides (nt) long non-coding RNAs, first revealed in eukaryotic organisms. Transcriptomic and transgenic methods have been employed to discover the functions of these microRNAs, as negative regulators of their target genes, during AR development in different plant species [34,35]. Individual miRNAs, however, cannot regulate AR formation; they must be associated with their target genes. For example, some auxin responsive factors, such as ARF17, ARF6, and ARF8, are regulated by miR160 and miR167 during AR development in *Arabidopsis* [36]; miR390 and ARFs form an auxin-responsive regulatory network that regulates lateral root initiation and growth from the main root [37]. In addition, a novel regulatory pathway, involving bidirectional cell signaling mediated by miR165 and miR166 and the transcription factors SHR (SHORT ROOT) and SCR (SCARECROW), has been recognized as establishing root cellular functions [38]. Hou et al. (2019) report that the overexpression of miR171 and miR390 in tomato plants can increase the lateral root number compared to wild-type plants [39]. Therefore, microRNAs are believed to be important regulators of plant growth and development. Elevated levels of miR156 promote AR development in tomato, tobacco, and maize [40]. Comprehensively, the formation of the root is a dynamic process that involves the integration of plant hormones, transcriptional regulators, and microRNAs to produce the correct AR formation [41].

Currently, clonal propagation through stem cuttings is the only way to deploy genetically improved tetraploid *R. pseudoacacia* L. varieties [7]. However, the AR mechanism, and key molecular factors that control AR formation under in vitro conditions, have not been fully explored. This study was intended to investigate the in vitro adventitious root formation of tetraploid *R. pseudoacacia* L. using dark-pretreated micro-shoot cuttings as plant materials. The main objectives of this study were to analyse (1) the effects of auxin on AR formation in dark-pretreated micro-shoot cuttings, (2) which genes are playing a role during IBA-dependent AR formation, while setting a particular focus on miRNAs and their interaction with putative target genes during the whole process of AR formation. To date, there is no investigation that describes how miRNAs and their targets modulate AR formation in tetraploid *R. pseudoacacia* L. Sequenced transcriptome and miRNA data were used to study the molecular mechanisms during etiolation-induced AR formation in tetraploid *R. pseudoacacia* L. It was anticipated that a better understanding of the underlying mechanisms in dark-pretreated micro-shoot cuttings will accelerate the genetic improvement of tetraploid *R. pseudoacacia* L. and provide the basis for further study on other tree species.

## 2. Materials and Methods

### 2.1. Plant Material, Growth Conditions, and Sample Collection

In the present study, tissue culture-grown micro-shoots (donor plants) of “tetraploid *R. pseudoacacia* L. clone-38” (recalcitrant clones) were used to study AR formation. Donor plants were propagated for four months in tissue culture medium in Murashige and Skoog (MS) [42], supplemented with 0.2 mg L^−1^ IBA, 30 g L^−1^ sugar, and 6 g L^−1^ agar at pH 5.8 in the laboratory at Beijing Forestry University, Beijing, China. Temperatures were maintained at 24 ± 1 °C with a 16/8-h photoperiod (photosynthetic photon flux density (PPFD) = 40–50 μmol m^−2^ s^−1^). These donor plants were used for the further experiment, in which donor plants underwent complete dark pretreatment for five days. Dark-pretreated donor plants were further divided into two categories, control (non-IBA) and treated (IBA), in which treated plants were supplemented with auxin IBA 0.2 mg L^−1^. Furthermore, lower portions, approximately 1–1.5 cm, of control and treated plants were evaluated to identify specific timing of AR formation after completion of five days of dark treatment. Treatment method was conducted according to Munir et al. [43], with slight modifications. On the basis of the specific timing of AR formation, samples for RNA-seq and miRNA-seq were collected from both non-IBA and IBA-treated micro-shoot cuttings and immediately stored in liquid nitrogen at −80 °C.

### 2.2. Paraffin Section Preparation and Microscopic Examination

At least 20 basal stem tissues (0.5 cm) were collected, with three replicates, for each time point from both control (non-IBA) and treated (IBA) plants to examine histological changes during AR formation at specific time points, i.e., 0, 12, 36, 48, and 72 h after excision (HAE). The collected tissues were fixed in formaldehyde acetic acid (FAA) solution (50% ethanol, 38% formaldehyde, 5% acetic acid) and treated as previously described [44]. Thin sections of 8 μm thickness were cut with a rotary microtome (LEICA RM2235) for histological observation. The cross-sections were placed on slides, stained with safranin (1%) and fast-green (0.5%) solution, and examined under a light microscope to observe the AR histology. All sections were photographed by LEICA DMI40008 microscope at the different time points.

### 2.3. RNA and Small RNA Library Construction and Sequencing 

The same methodology was applied to collect samples for miRNA-seq and RNA-seq as that conducted for the collection of histological samples from the basal portion (0.5 cm) of tetraploid *R. pseudoacacia* L. micro-shoot cuttings. 

First, total RNA extraction (15 samples) from the cuttings at the time of excision (0 HAE) and of the IBA treatment of “tetraploid *R. pseudoacacia* L. clone-38” micro-shoot cutting bases collected at 12, 36, 48, and 72 HAE, was carried out according to the protocol described by Munir (2021) [43], in which RNA purity (OD260/280) was ensured using ultraviolet spectrophotometer Nanodrop.

Total miRNA was also extracted from the basal portion of “tetraploid *R. pseudoacacia* L. clone 38” micro-shoot cuttings (0.5 cm) at the above-mentioned time points. After that, seven different comparison libraries (0-vs-12, 0-vs-36, 0-vs-48, 0-vs-72, 12-vs-36, 36-vs-48, and 48-vs-72 HAE) were constructed for RNA and miRNA sequencing. The final quality of the cDNA library was ensured using an Agilent2100 Bioanalyzer (Agilent Technologies, Santa Clara, CA, USA). RNA was fractionated on a 15% denaturing polyacrylamide gel. The miRNA regions corresponding to 18–30 nt were excised and recovered. These sRNAs were then 5’ and 3’ RNA adapter-ligated using T4 RNA ligase (Takara, Dalian, China). Ligated products were purified using an Oligotex mRNA mini kit (Qiagen, Hilden, Germany) and subsequently transcribed into cDNAs via a SuperScript Ⅱ RT (Invitrogen, Carlsbad, CA, USA). PCR amplifications were performed with primers annealed to the ends of the adapters.

### 2.4. Analysis of Differentially Expressed miRNAs and Their Target Genes

To further reveal the possible functions of differentially expressed (DE) miRNAs and their associated target genes, all target genes were investigated against Gene Ontology (GO) and Kyoto Encyclopedia of Genes and Genomes (KEGG) databases (http://www.genome.jp/kegg/, accessed on 15 August 2021). FDR ≤ 0.05 was the threshold for significant enrichment in GO and KEGG enrichment analyses. The expression profiles of the differentially expressed miRNAs and their target genes were visualized with TBtools software, Guangdong, China [45].

### 2.5. miRNA Identification during AR Formation

Tag sequences derived from deep sequencing were treated via Phred and Cross-match (http://www.phrap.org/phredphrapconsed.html, last accessed on 30 November 2020) in which clean reads were obtained by the removal of adapter sequences; alternatively, reads in which the presence of unknown base N was greater than 5%, and low-quality sequences (in which the percentage of low-quality bases with quality value ≤ 10 was greater than 20%) were filtered, and low-quality tags and contamination were removed from adaptor sequences not ligated to any other sequences. The high-quality miRNA reads were then trimmed from their adapter sequences. scRNAs, snRNAs, rRNAs, tRNAs, and snoRNAs were detached from the miRNA sequences by BLASTn search using the NCBI Genbank database (http://www.ncbi.nlm.nih.gov/blast/Blast.cgi/ accessed on 30 November 2020) and Rfam (11.0) database (http://www.sanger.ac.uk/resources/databases/rfam.html accessed on 30 November 2020). Using tag2 annotation, all annotations were summarized by BGI software using the following series of preferences: rRNA (Genbank > Rfam3) > known miRNA > piRNA > repeat > exon > intron. From miRbase22 (http://www.birbase.org/, accessed on 30 November 2020) with up to two mismatches, the residual sequences were aligned to known plant miRNAs. Mireap (http://sourceforge.net/projects/mireap/, accessed on 30 November 2020) software package developed by BGI through screening the biological characteristics of miRNAs was used to predict novel miRNAs that were not annotated.

### 2.6. Differential Expression (DE) Analysis of miRNAs

In accordance with the expression of transcript per million (TPM) to the total clean miRNA reads in each sample, the frequencies of each known miRNA read count were normalized. The fold change of miRNA expression among individual treated (IBA) and control (non-IBA) plants was calculated as log2 (treated/control). Providing there was a significant difference in expression between the two samples, the DESeq2 package (Washington, DC, USA) was used. Under the conditions that *p* ≤ 0.05, with a normalized seq-count log2 (treated/control) > 1 or <−1, the specific miRNA was considered differentially expressed (DE). 

### 2.7. Target Prediction of miRNAs

TargetFinder (http://github.con/carringtonlab/TargetFinder, Santa Clara, CA, USA, accessed on 15 August 2021) and psRNA Target software package (http://plantgrn.nobe.org/psRNATarget/ Ardmore, OK, USA, accessed on 15 August 2021) were used for target prediction of miRNAs; for candidate target prediction, default parameters were designated. Genes that overlapped among the two software applications were considered as target genes. Subsequently, enrichment analysis was performed using GO terms and KEGG pathways. Selected miRNAs and their target genes’ co-expression networks were visualized by Cytoscape software package (v3.7.2, Boston, CA, USA).

### 2.8. Relative Expression Analysis miRNA and Their Targets by Quantitative Real-Time PCR (qRT-PCR)

In order to quantify the uncovered miRNAs and mRNAs, poly(A) extension quantitative real-time polymerase chain reaction (qRT-PCR) was carried out using a previously established protocol, with slight changes [46]. All the qRT-PCR templates were created using 3 μg total RNA isolated from the control, and treated at 0, 12, 36, 48, and 72 HAE. The qRT-PCR reactions were performed using Mini Opticon Real-Time PCR System (Bio-Rad, Hercules, CA, USA). The normalized apple actin gene was used as a reference standard in the analysis [47]. SYBR Green Talent qPCR PreMix TianGen RNAprep Pure Plant Kit (TIANGEN, Beijing, China) was used according to protocol. The relative expressions of miRNAs and their targeted genes were calculated according to the formula 2^−ΔΔCt^, as previously reported by Min et al. [48]. Each reaction was executed with three biological replicates, and each sample was analyzed in triplicate (technical replicates). Mean expression and standard error (SE) were calculated from the results of three independent replicates. Graphs were generated and statistically evaluated using Mev 4.0 software (http://www.tm4.org/, Boston, CA, USA, accessed on 15 August 2021) and *p* < 0.05 was the significance threshold. The particular qRT-PCR primer sequences for selected target genes and miRNAs were designed (Supplementary Table S6).

## 3. Results

### 3.1. The Effect of IBA on the Development of AR Formation in Dark-Pretreated Cuttings of Tetraploid R. pseudoacacia

**Figure 1:**
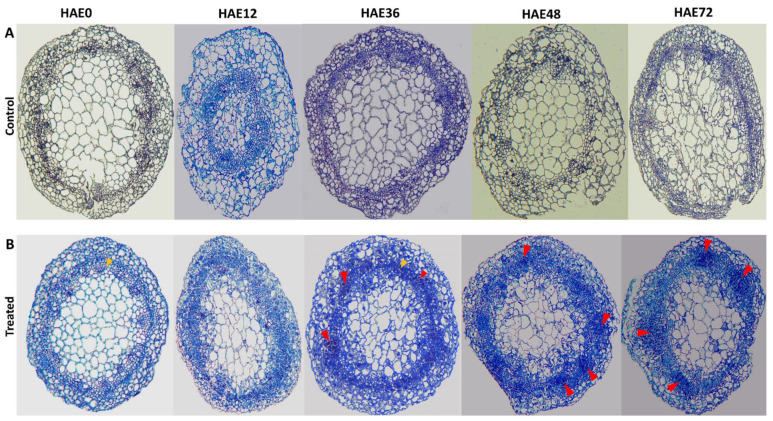
Histological observations of dark-pretreated micro-shoot cuttings (IBA and non-IBA) of tetraploid *R. pseudoacacia* L. during AR formation process at 0, 12, 36, 48, and 72 HAE, in which only treated micro-shoots were subjected to 0.2 mg L^−1^ IBA. (**A**) Histological observations of control (non-IBA), dark-pretreated micro-shoot cuttings, with no changes at mentioned HAE. (**B**) Histological observations of treated (IBA), dark-pretreated micro-shoot cuttings with apparent changes at HAE. Yellow arrows indicate the cambium layer, red arrows represent the first mitotic cell divisions and first AR primordium development on the cambium layer.

The histological changes during the entire AR formation process were observed under in vitro growth conditions (25 °C with a 16/8-h photoperiod). In order to further observe the AR development in dark-pretreated micro-shoot cuttings (non-IBA and IBA), histological analyses were carried out using paraffin sections before the emergence of AR primordia through the cuticle layer (within 72 h) in control (non-IBA) and treated (IBA) micro-shoot cuttings. No cellular activities were observed in the control micro-shoot cuttings during the overall process of AR formation within 72 h (Figure 1A). However, the first cell division was observed at 36 HAE (Figure 1B) when treated micro-shoot cuttings were immediately exposed to auxin-containing media. It was observed that there was a significant proportion of cells with high ploidy levels (maximum cell division increases the number of cells) on the cambium layer at 36 HAE (Figure 1B). Moreover, these structures increased further during the subsequent rooting process at 48 HAE (Figure 1B). The primary AR meristems began to develop after 48 HAE, and several root primordia were formed after 72 HAE (Figure 1B). Therefore, only the 0 HAE time point was selected from control micro-shoot cuttings (non-IBA), as no histological observations were made at the other time points for the control cuttings. Consequently, five key time points of treated and control micro-shoot cuttings (0, 12, 36, 48, and 72 HAE) were used for further analysis of miRNA expression profiling during AR development.

### 3.2. Identification of DEGs and MicroRNAs during Dark-Pretreated IBA-Dependent AR Formation in Tetraploid R. pseudoacacia L.

The metabolic variations in plants are regulated by many factors, including the microRNA-mediated degradation of mature RNA. Five comparison libraries (HAE0, HAE12, HAE36, HAE48, and HAE72) were constructed to analyse gene and miRNA expression. The data generated by RNA-seq were able to achieve 100% tetraploid *R. pseudoacacia* L. genome coverage. Additionally, we perceived a high correlation value among the replicates in each sample (Figure S1). To determine the temporal changes during AR development in the dark-pretreated micro-shoot cuttings, the samples already described in the context of RNA extraction were compared regarding mRNA and miRNA abundance. Therefore, we constructed mRNA and microRNA (miRNA) libraries using the tetraploid *R. pseudoacacia* L. micro-shoot cuttings collected from the aforementioned five time points of AR developmental stages. A total of 671.683 million clean reads were generated from the 15 libraries (Table S1). After removing the adapter sequences, short reads, and low-quality reads, the unique sRNAs were annotated using the Rfam database to exclude small nuclear RNA (snRNA), small nucleolar RNA (snoRNA), and ribosomal RNA (rRNA) sequences (Figure S2).

To identify the microRNAs related to AR formation in dark-pretreated micro-shoot cuttings, 15 samples, including three biological replicates per time point, were prepared for RNA sequencing. The numbers of raw sequencing reads per sample that ranged from 8.6 to 11.1 million were obtained for the four time points, HAE12, HAE36, HAE48, and HAE72, and an average of 9.8 million reads were obtained for the control samples (HAE0) (Table S2). After removing adaptor and low-quality reads, at least 5.5 million clean reads were obtained for each sample (Table S2). Between 3.1 and 3.4 million reads were aligned to the tetraploid *R. pseudoacacia* L. genome, with a mapping rate that ranged from 59.94% to 62.92%. A total of 674 known miRNAs were identified, and 1099 novel miRNAs were predicted across all libraries. Novel microRNAs were all found to possess hairpin structures and star sequences (Table S3). After removing the low-quality sequences, the lengths of miRNAs ranging from 18 to 30 nt were screened for subsequent analysis. The size distribution of the miRNAs was generally the same, and the sizes of the 21 and 24-nt miRNAs were extremely redundant among all seven comparison libraries (Figure S2). In order to characterize the microRNAs, the length distributions of the miRNAs (known and novel) were analyzed and plotted (Figure S2). Among these known miRNAs, the most abundant reads were found to be 21 nt in length, and the most abundant novel miRNAs had a sequence of 24 nt (Figure S2). The dominance of the 24 nt long sequences suggested that they were small interfering RNA (siRNA) [49], which is consistent with previous studies from other species, including *Capsicum chinense* [50], *Solanum lycopersicum* [51], *Arabidopsis thaliana* [52], sorghum [53], *Oryza sativa* [54], *Prunus avium* L. [55] and *Zea mays* [56]. This suggested that more post-transcriptional modifications might exist in tetraploid *R. pseudoacacia* L. micro-shoot cuttings, as the 24-nt miRNAs form the majority of small interfering RNAs [57]. All the clean reads obtained were compared against the Rfam database. We observed that most of the clean reads (more than 70%) belonged to unannotated miRNAs, and only ~0.5–1% of clean reads belonged to miRNAs in the five comparison libraries (Figure S2).

The analysis of the nucleotide components for these miRNAs showed that the number of nucleotides at different cleavage sites of the miRNA sequences was altered in the HAE0, HAE12, HAE36, HAE48, and HAE72 libraries. Further analysis of 1–24 nucleotides revealed that the lowest and highest number of nucleotides were for guanine and cytosine, respectively, in all libraries except HAE72, where adenine exhibited the lowest number (Figure S2).

### 3.3. Comparative Analysis of DEGs, MicroRNAs, and Their Expression Profiles during IBA-Dependent AR Development in Dark-Pretreated Cuttings of Tetraploid R. pseudoacacia L.

**Figure 2:**
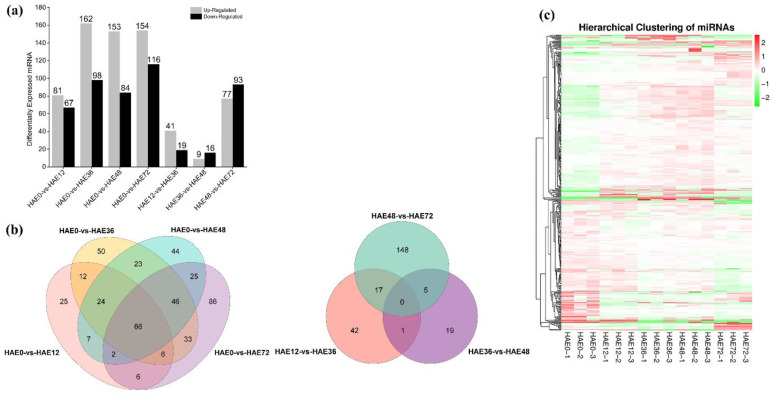
The number of differentially expressed (DE) microRNAs in dark-pretreated micro-shoot cuttings of tetraploid *R. pseudoacacia* L. during IBA-dependent AR formation in seven comparison group libraries (HAE0-vs-HAE12, HAE0-vs-HAE36, HAE0-vs-HAE48, HAE0-vs-HAE72, HAE12-vs-HAE36, HAE36-vs-HAE48, and HAE48-vs-HAE72). (**a**) Total upregulated and downregulated DE miRNAs expressed in seven comparison group libraries. (**b**) Venn diagrams showing the number of the differentially expressed miRNAs in the seven comparison group libraries. (**c**) Heatmap showing the fold changes of miRNAs in comparison group libraries.

**Figure 3:**
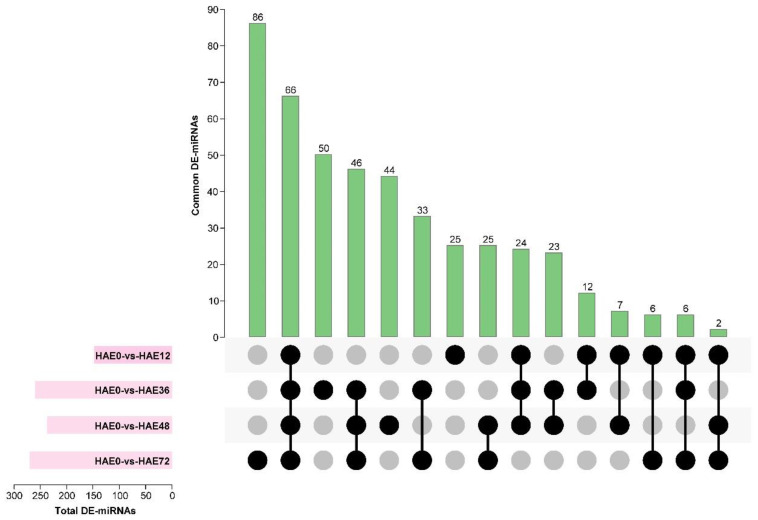
Expression profile and identification of DE miRNAs during IBA-dependent AR formation in dark-pretreated micro-shoot cuttings of tetraploid *R. pseudoacacia* L. The figure depicts the shared and unique (known and novel) DE miRNAs of the associated transcriptome analyses. The overlap between the DE miRNAs was identified following 0 HAE, 12 HAE, 36 HAE, 48 HAE, and 72 HAE from the donor plant. The *y*-axis shows the number (0–90) of miRNA interactions. The *x*-axis represents the total number of miRNAs expressed in the first four comparison group libraries. Black circles show the common DE miRNAs in different comparison groups.

Based on identifying differentially expressed (DE) microRNAs, we constructed seven comparison group libraries for miRNAs involved in the early hours of AR development. We depicted enhanced expression of 81, 162, 153, 154, 41, 9, and 77 miRNAs, and decreased expression of 67, 98, 84, 116, 19, 16, and 93 miRNAs, in the HAE0-vs-HAE12, HAE0-vs-HAE36, HAE0-vs-HAE48, HAE0-vs-HAE72, HAE12-vs-HAE36, HAE36-vs-HAE48, and HAE48-vs-HAE72 comparison group libraries, respectively (Figure 2a and Table S5). The Venn diagrams (Figure 2b and Figure 3) revealed that the 66 differentially expressed (DE) microRNAs were presented in all groups (HAE0-vs-HAE12, HAE0-vs-HAE36, HAE0-vs-HAE48, and HAE0-vs-HAE72), meaning that the 66 miRNAs were involved during the whole initial phase of AR primordia development. In contrast, no DE miRNA was expressed in the consecutive comparison group (HAE12-vs-HAE36, HAE36-vs-HAE48, and HAE48-vs-HAE72). On the other hand, 25, 50, 44, 86, 42, 19, and 148 miRNAs were only implicated in the initial hours of the AR primordia development process, respectively. Moreover, the microRNAs in the HAE0-vs-HAE12, HAE0-vs-HAE36, HAE0-vs-HAE48, HAE0-vs-HAE72, HAE12-vs-HAE36, HAE36-vs-HAE48, and HAE48-vs-HAE72 comparison libraries unveiled a less than five-fold change in expression (Figure 2c).

Among the seven comparison groups of miRNA libraries, we analyzed the sequence data of only four (HAE0-vs-HAE12, HAE0-vs-HAE36, HAE0-vs-HAE48, and HAE0-vs-HAE72) comparison group libraries for further analysis, as there was no significant difference between the consecutive comparison groups of the miRNA libraries in the dataset.

**Figure 4:**
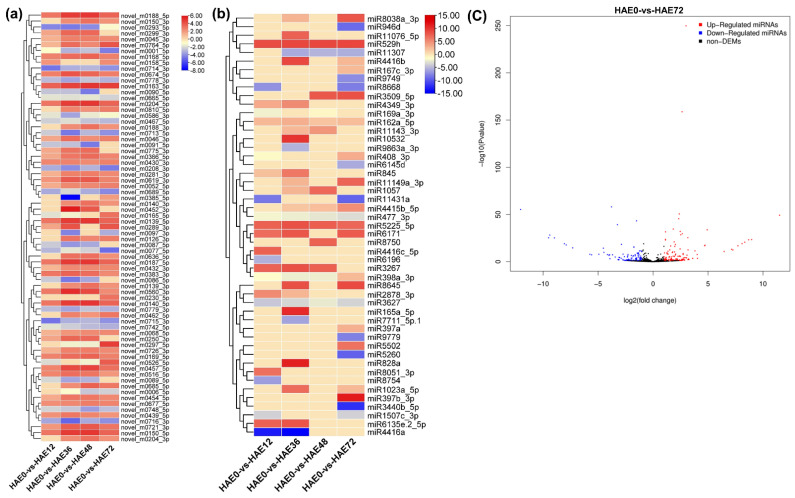
Hierarchical clustering of differentially expressed miRNAs in dark-pretreated tetraploid *R. pseudoacacia* L. during IBA-dependent AR formation. (**a**) Heatmap showing the number of the known differentially expressed upregulated and downregulated miRNAs in the four comparison groups. (**b**) Heatmap showing the expression pattern of the novel differentially upregulated and downregulated miRNAs. (**c**) A volcano plot showing the DE miRNAs in the HAE0-vs-HAE72 comparison group libraries. The clusters were generated based on the Pearson correlation coefficient of normalized miRNA expression values. Bars indicate log2 (TPM + 1) values.

Several known and novel miRNA family members were identified in all four comparison group libraries (Figure 4a,b), including miR529h and miR5225-5p, and were highly differentially expressed in all four comparison groups; alternatively, miR6171 and miR3267 were highly expressed in all comparison groups except HAE0-vs-HAE48 and HAE0-vs-HAE72, respectively, in the known miRNAs. The expression level of miR529h increased sharply from HAE0-vs-HAE12 to HAE0-vs-HAE72. On the other hand, miR5225-5p expression showed an increasing trend at HAE0-vs-HAE36; however, it fluctuated at HAE0-vs-HAE12 and HAE0-vs-HAE48, and showed a decreasing trend at HAE0-vs-HAE72. The abundance of miR6171 and miR3267 increased during the early hours of AR development, but decreased in the late hours (HAE0-vs-HAE48 and HAE0-vs-HAE72), respectively, whereas was miR162a-5p expressed during the whole induction phase of AR development. The miR397b-3p exhibited higher expression at HAE0-vs-HAE72, and was suppressed from HAE0-vs-HAE12 to HAE0-vs-HAE48 (Figure 4a).

Whereas novel miRNAs, such as miR0163-5p, miR0139-5p, miR0187-5p, miR0150-5p, miR0188-5p, miR0140-5p, and miR0204-5p, were highly abundant in all four comparison group libraries, the abundance of miR0163-5p fluctuated at HAE0-vs-HAE12; however, it showed high expression at HAE0-vs-HAE36 to HAE0-vs-HAE72, respectively. Furthermore, miR0139-5p, miR0187-5p, miR0150-5p, miR0188-5p, miR0140-5p, miR0204-5p expression fluctuated at HAE0-vs-HAE12 and HAE0-vs-HAE72, with increasing trends at HAE0-vs-HAE36 and HAE0-vs-HAE48 (Figure 4b). The volcano plot presented in Figure 4c displays the DE miRNAs in the HAE0-vs-HAE72 group, which exhibited more than 150 upregulated, and 100 downregulated, miRNAs.

### 3.4. Annotation of Genes and MicroRNA during IBA-Dependent AR Formation in Dark-Pretreated Cuttings of Tetraploid R. pseudoacacia L. 

**Figure 5:**
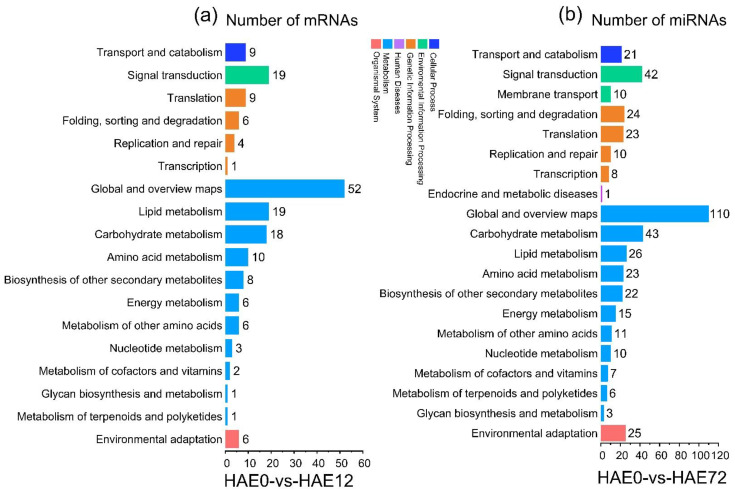
KEGG classification of differentially expressed genes (DEGs) and differentially expressed microRNAs in HAE0-vs-HAE12 and HAE0-vs-HAE72 comparison libraries. (**a**) The number of DEGs in HAE0-vs-HAE12 libraries. (**b**) The number of DE miRNAs in HAE0-vs-HAE72 libraries. The *x*-axis represents the number of DEGs and DE miRNAs. The *y*-axis shows the KEGG pathway terms in DEGs and DE miRNAs.

Based on the differentially expressed genes (DEGs) in HAE0-vs-HAE12, HAE0-vs-HAE36, HAE0-vs-HAE48, and HAE0-vs-HAE72, all DEGs were clustered into five groups, presented as heatmaps in Figure S3. We annotated the DEGs and miRNAs in the HAE0-vs-HAE12, HAE0-vs-HAE36, HAE0-vs-HAE48, and HAE0-vs-HAE72 comparison group libraries during AR formation using the KEGG tool. All the DEGs and miRNAs were classified into six groups; cell processes, environmental information processing, genetic information processing, human diseases, metabolism, and organismal systems. In the case of the gene expression comparison libraries, we observed that most pathway changes were associated with global and overview maps (52, 103, 83, and 110 genes with altered mRNA level), followed by signal transduction (19, 32, 37, and 42 DEGs), and carbohydrate metabolism (18, 34, 23, and 43 DEGs) in the HAE0-vs-HAE12, HAE0-vs-HAE36, HAE0-vs-HAE48, and HAE0-vs-HAE72 comparison libraries (Figure 5 and Figure S4). 

KEGG pathway enrichment analysis was performed based on the DEGs. Figures S5–S7 indicates 26 enriched KEGG metabolic pathways during AR formation, in which a greater level of enrichment occurred in the initial hours of AR developmental stages, i.e., HAE0 to HAE12. Among them, two metabolic pathways, including plant hormone signal transduction and starch and sucrose metabolism, were selected for further analysis (Figures S5–S7). Moreover, 78 and 20 DEGs were enriched in the plant hormone signal transduction and starch and sucrose metabolism pathway. The KEGG pathway results showed differences in gene expression in plant hormone signaling and starch and sucrose metabolism, indicating their independent or joint actions in the AR development process. According to sequence homology, various miRNAs and their target genes were identified that were known to be potentially involved in the AR formation process. These included genes associated with auxin biosynthesis and signal transduction, i.e., auxin-inducible gretchen hagen3 (*GH3-9*), small auxin upregulated RNA-70 (*SAUR70*), and auxin response transcription factor-3 (*ARF3*), ethylene biosynthesis and ethylene signaling and response pathways, i.e., ethylene response 2 (*ETR2*), and gibberellin biosynthesis and signal transduction pathways, i.e., scarecrow-like-5 (*SCL5*). Additionally, genes associated with brassinolide signaling pathways included squamosa promoter-binding protein-like-12 (*SPL12*) and Salicylic Acid transcription factor-*9* (*TGA9*). Moreover, miRNA encoding several key genes involved in cell wall metabolism, carbohydrate transport, and metabolism during AR development included ADP glucose pyrophosphorylase large subunit (*APL4*), vacuolar invertase-1 (*VI1*), starch synthase-3 (*SS3*), and probable starch synthase-3 (*SPS3*).

### 3.5. Association Analysis of miRNAs and Targeted Genes during IBA-Dependent AR Formation in Dark-Pretreated Cuttings

**Figure 6:**
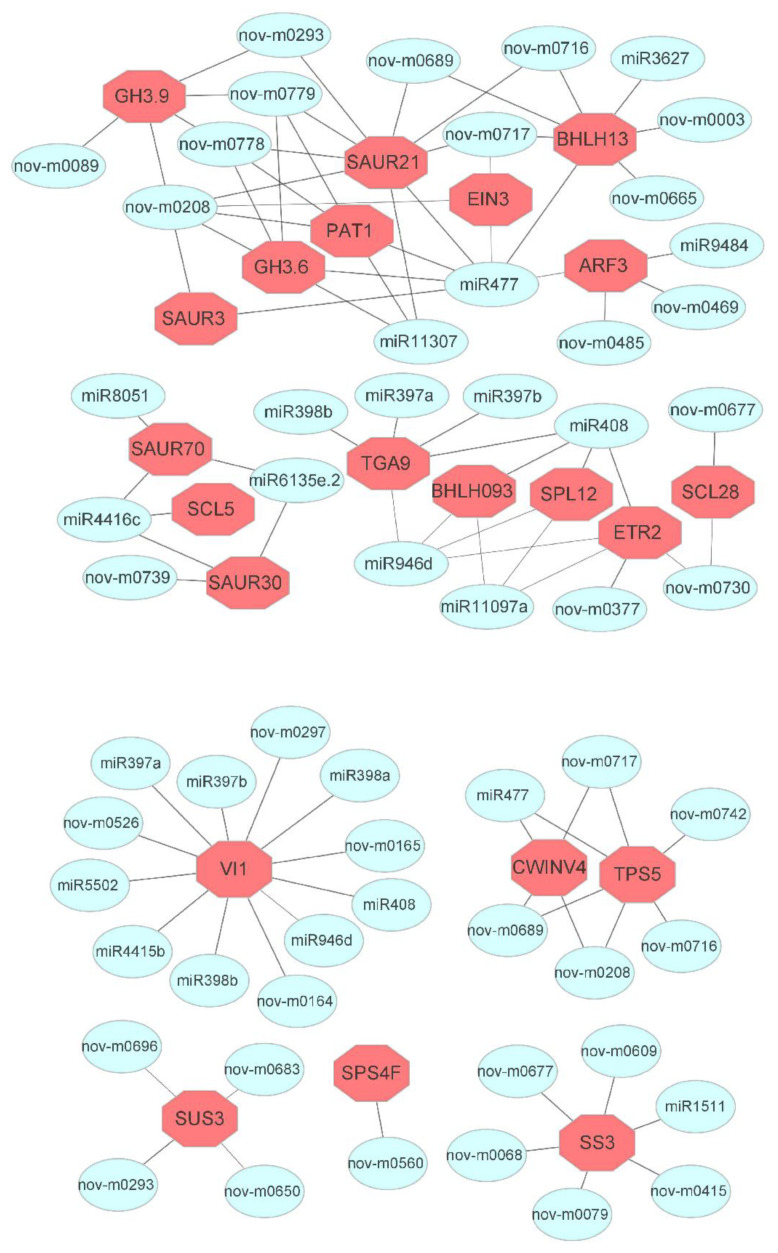
Co-expression network of the miRNAs and their targeted genes expressed in dark-pretreated micro-shoot cuttings of tetraploid *R. pseudoacacia* L. during IBA-dependent AR formation. Co-expression network of miRNAs and their targets during plant hormone signal transduction and sucrose metabolism pathways at five time points.

We carried out the association analysis to evaluate the correlation between DEGs and miRNAs during AR development. To further study the potential roles of the DE miRNAs and their target genes in the regulation of AR development, target genes were predicted using the psRNATarget program (Table S4). Based on the function of the functional category annotations, correlations between miRNAs and their target genes were outlined (Figure 6).

### 3.6. MicroRNAs and Targeted Genes Involved during Sucrose Metabolism and Hormone Signaling Transduction Pathways

We selected 15 DEGs in the plant hormone signal transduction pathway (*BHLH13*, *BHLH093*, *SAUR3*, *SAUR 21*, *SAUR 70*, *SAUR 30*, *ARF3*, *GH3.9*, *GH.3.6*, *PAT1*, *SCL5*, *SCL28*, *SPL12*, *TGA9*, *EIN3*, and *ETR2*) and their corresponding 30 DE miRNAs. In comparison, six DEGs were selected by analyzing starch and sucrose metabolism pathways (*SPS4F*, *SUS3*, *CWINV4*, *VI1*, *SS3*, and *TPS5*), including their corresponding 32 DE miRNAs (Figure 6). 

We predicted that miR477-3p would target *BHLH13* (gene10048), *SAUR3* (gene16759), *ARF3* (gene13686), *PAT1* (gene24841), *SAUR21* (gene16750), *EIN3* (gene1693), and *GH3.6* (gene24554) genes, including other corresponding miRNAs (6, 1, 3, 4, 8, 2, 4), respectively. Five miRNAs, including novel-m0089-5p targeting *GH3.9* (gene19436), miR4416c-5p targeting *SCL5* (gene15254); four miRNAs, including miR11097a-5p targeting *SPL12* (gene15549); six miRNAs, including miR397b-3p targeting *TGA9* (gene16116); three miRNAs including miR4416c-5p targeting *SAUR70* (gene34995); two novel miRNAs, including novel-m0677-3p targeting *SCL28* (gene1179); miR4416c-5p and two others targeting *SAUR30* (gene25953), and finally miR11097a-5p and two others targeting *BHLH93* (gene35754). Furthermore, five miRNAs, including miR11097a-5p, were predicted to target *ETR2* (gene17473) genes during the phytohormone signal transduction pathway. On the other hand, when analyzing the starch and sucrose metabolism pathway, the *SPS4F* (gene25748) gene was predicted to be modulated by novel miR0560-3p. The *SUS3* (gene9499) gene was shown to be regulated by four novel miRNAs, including novel-miR0293-5p. The *CWINV4* (gene21632) and *TPS5 9* (gene36418) genes were the possible target genes of miR477-3p and their other corresponding miRNAs (3 and 5), respectively. In addition, *VI1* (gene30969) was found to serve as the target gene of 14 miRNAs, including miR397a. Furthermore, *SS3* was predicted to act as the target gene of six miRNAs, including miR1511 (Figure 6).

### 3.7. Relative Expression Analysis of Differentially Expressed miRNAs and Their Targeted Genes by qRT-PCR Assay

**Figure 7:**
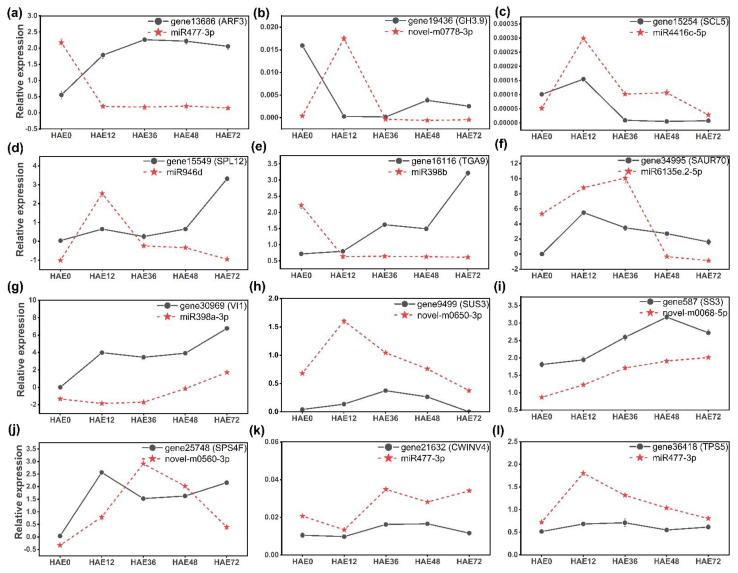
Expression analysis of ten miRNAs and their twelve target genes obtained from dark-pretreated micro-shoot cuttings of tetraploid *R. pseudoacacia* L. at 0, 12, 36, 48, and 72 HAE. (**a**–**f**) The genes and their respective miRNA expression during the auxin signaling pathways. (**g**–**l**) The genes and their respective miRNA expression during the starch and sucrose metabolism pathway. The data presented are an average of three technical replicates.

The expression patterns of key genes and their respective miRNAs were further validated by the qRT-PCR analysis. These patterns played a critical role during IBA-dependent AR formation in dark-pretreated micro-shoot cuttings of tetraploid *R. pseudoacacia* L. The genes and miRNAs of enriched pathways, plant hormone signal transduction, and starch and sucrose metabolism pathways were analyzed to confirm their role and consistency with the results of RNA-seq data. Figure 7 depicts the relative expression profiles of 12 miRNAs (four novel and eight conserved) and their corresponding target genes at 0, 12, 36, 48, and 72 HAE. The qRT-PCR analysis confirmed that the RNA-seq results and relative expression patterns of the 12 targeted genes, with their respective 10 microRNAs, were consistent.

Among the plant hormone signaling pathways, *GH3.9, SAUR70, ARF3, SCL5, SPL12,* and *TGA9* genes played a critical role during AR formation. The most important *ARF3* (gene-13686) showed differential expression, in which gene expression increased from 0 HAE to 72 HAE; the expression of respective miRNA (miR477-3p) followed the opposite trend (Figure 7a). Meanwhile, expression, in the case of *SAUR70* (gene-34995), decreased continuously after 12 HAE, and the expression of respective miRNA (miR6135e.2-5p) increased from 0 HAE to 36 HAE, then continuously decreased (Figure 7f). Additional to these genes, the expression of *SCL5* (gene-15254) and its respective miRNA (miR4416c-5p) was highest at 12 HAE (Figure 7c). Moreover, *SPL12* and *TGA9* also played a critical role during AR formation, in which the expression of *TGA9* (gene-16116) and its respective miRNA (miR398b) was the same at 12 HAE, whereas *TGA9* expression continuously increased up to 72 HAE, and miRNA expression remained almost the same up to 72 HAE (Figure 7e). Thus, all hormone-related genes and their respective miRNAs showed consistent regulation during IBA-dependent AR formation in dark-pretreated micro-shoot cuttings of tetraploid *R. pseudoacacia* L. 

Furthermore, during sucrose biosynthesis, vacuolar invertase-1 (*VI1*) and starch synthase-3 (*SS3*) gene expression levels were significantly upregulated during different stages of AR formation, which corresponded to the lower expression of their respective miRNAs (miR389a-3p and novel-m0068-5p) (Figure 7g,i). Sucrose synthase (*SUS3*) expression increased up to 36 HAE, and subsequently decreased over time to 72 HAE, whereas its respective novel miRNA (novel-m0650-3p) increased at 12 HAE and then continuously decreased during AR formation (Figure 7h). Moreover, the expression of probable starch synthase-3 (*SPS3*) increased from 36 HAE to 72 HAE, while the expression of its respective miRNA (novel-m0560-3p) showed the opposite trend (Figure 7j). Furthermore, the functional relationships between the miRNAs and their respective genes were not analyzed for these cuttings. Therefore, both cell wall invertase-4 (*CWINV4*) and trehalose phosphatase synthase-5 (*TPS5*) gene expressions were downregulated by the same miRNA (miR477-3p). In the first case (*CWINV4*), expression was highest at 36 HAE, then continuously decreased over time to 72 HAE; in second case (*TPS5*), highest expression was at 12 HAE, after which it decreased continuously (Figure 7k,l). The differential expression of these miRNAs and their respective target genes controlling the carbohydrate metabolism indicate that they contribute to the IBA-dependent adventitious root formation in dark-pretreated micro-shoot cuttings of tetraploid *R. pseudoacacia* L.

## 4. Discussion

### 4.1. Anatomical Observations during IBA-Dependent AR Formation in Dark-Pretreated Micro-Shoot Cuttings

The AR formation in the etiolated micro-shoot cuttings of tetraploid *R. pseudoacacia* L. was dependent on exogenous IBA application. In the present study of dark-pretreated micro-shoot cuttings of tetraploid *R. pseudoacacia* L., the earliest anatomical observations from 36 HAE to 48 HAE were unambiguously related to AR development, whereas initiation was observed at 72 HAE. (Figure 1). Our findings indicate cell division in the cambial cells near the xylem of the dark-pretreated micro-shoot cuttings, which defines the induction phase of AR development [31,58]. In addition, increased cell division was observed in the vascular cambium and the adjacent tissues, specifically in the parenchyma and phloem cells region (Figure 1). The dark pretreatment of the donor plants accelerates the formative cell division in the cambium, leading to the formation of AR primordia; the AR initiation phase is critical for AR formation [59]. Some meristemoids become true root primordia (Figure 1), subsequently grow through the surrounding layers of tissue, and develop a vascular system during the expression phase [60]. Later, the tissue organization of the root primordia and their vascular connection with the stem is established. The developmental phases of AR have been described previously in apple cuttings [61], and similar sequential phases were also reported in other tree species [62,63].

### 4.2. Identification and Expression Profiling of miRNAs and Their Targets during IBA-Dependent AR Formation in Dark-Pretreated Tetraploid R. pseudoacacia L.

Illumina RNA-seq technology is an efficient approach for exploring novel miRNA, and is extensively used for model plant transcriptome and miRNA sequencing [64], both with reference genome data and non-model plants without genomic reference information [65]. It has been extensively used to analyse the molecular regulation of plant growth and development at a transcriptional level [66,67]. We examined the global gene expression of control (non-IBA) and treated (IBA) dark-pretreated micro-shoot cuttings up to 72 HAE. To identify the expression of miRNAs and their target genes, we constructed seven differentially expressed comparison libraries (0-vs-12, 0-vs-36, 0-vs-48, 0-vs-72, 12-vs-36, 36-vs-48, and 48-vs-72 HAE), using the Illumina Hi-Seq 2000 platform, (GENECODE, Beijing, China) to perform a de novo microRNA sequencing analysis of the tetraploid *R. pseudoacacia*, in order to better understand the expression changes during AR formation. Pooled RNA samples from treated and control dark-pretreated micro-shoot cuttings sampled at five time points after inoculation in auxin medium were used to construct cDNA libraries for deep sequencing. Table S1 depicts that, in total, 671.683 M paired-end clean reads were generated, with a mean length of 24 nucleotides (Figure S2) [65,68,69]. We detected miRNAs between seven comparison groups [70], and also screened many differentially expressed genes during different stages of AR formation [71]. In addition, some miRNAs exhibiting altered expression in 0-vs-12, 0-vs-36, 0-vs-48 and 0-vs-72 were selected (Figure S2; Tables S4 and S5). The results obtained in the current study indicate a significant difference in expression of miRNAs during adventitious initial rooting stages, suggesting that the mechanism of miRNA regulation might be different between different stages.

Based on the enriched pathway analysis, we identified that starch and sucrose metabolism and plant hormone transduction were highly enriched during AR formation, and might play a role in dark-pretreated IBA-dependent AR formation. We discovered that essential miRNAs and their target genes play a role in AR by exogenous IBA application. The above-mentioned miRNAs and genes associated with these pathways, and their corresponding RT-qPCR results, are described in their respective results section.

### 4.3. miRNAs and Their Target Genes during Plant Hormone Signaling Transduction Pathway 

To date, several genes that regulate adventitious rooting have been uncovered in woody species. However, the role of miRNAs in tetraploid *R. pseudoacacia* L. AR formation has not been systemically explored [72]. The present study identified hormone signaling pathway-related miRNAs and their target genes [73]. A number of miRNAs have been recognized to regulate root growth by targeting various genes during AR root formation [33,74]. For example, miR156 is necessary for lateral root growth by targeting its target genes *SPL9* and *SPL10* [75], whereas miR160 modulates root development by the negative regulation of its target genes *ARF10*, *ARF16*, and *ARF17* [76]. Currently, it has been shown that miR396 regulates the stem cell niche by cutting GRFs, and thus regulates cell division in *Arabidopsis* roots [77]. It appears, from our data, that these microRNAs play a critical role during IB-dependent AR formation in dark-pretreated micro-shoot cuttings of tetraploid *R. pseudoacacia* L.

The KEGG enrichment analysis discovered that the genes targeted by microRNAs are mostly enriched in phytohormone signaling pathways. Therefore, many miRNAs and mRNA modules associated with phytohormone signaling were revealed. These modules included miR477-3p_*ARF3*, novel-m0778-3p_*GH3.9*, and miR6135e.2-5p_*SAUR70*, which are involved in the auxin signaling pathway. Generally, the upregulation of miRNA leads to the downregulation of their target genes, and vice versa [78]. In many plant species that are hard to root, the auxin analog IBA stimulates adventitious rooting [79], as was found in our study. Our results indicate that the auxin-inducible gretchen hagen-3 gene, *GH3.9*, may function in the conjugation of IBA or derived IAA to amino acids, because GH3 proteins function as acyl acid amido synthases. These GH3 homologs are acknowledged as being required for fine-tuning AR initiation in the etiolated hypocotyls of *A. thaliana* [80]. However, in woody plants, such knowledge is currently limited [81]. Here we observed that GH3 relative expression was negatively correlated with novel-m0778-3p [82]. The relative expression of auxin response factor-3 (*ARF3*) was significantly expressed in all samples at the respective time points compared to controls due to the lower negative expression of miRNA477-3p [35,83].

Furthermore, *SAUR70* was downregulated at 12 up to 36 HAE, and slightly upregulated at 48 up to 72 HAE at the initiation phase, due to the high negative expression and reduced negative expression of miR6135e.2-5p, respectively (Figure 7). However, a previous report also indicated that dark treatment induced the expression of many *SAUR* genes, which may be involved in dark-mediated AR induction [84]. Similar to auxin, brassinolide (BL) also has positive effects on root development [85]. The relative expression of the squamosa promoter-binding protein-like-12 gene, *SPL12*, which may be regulated by light and phytohormones [86], was significantly upregulated at the early hours of the initiation phase due to the lower negative expression of its corresponding miR946d (48–72 HAE); however, at the initial hours (12 HAE) it showed lower expression because of the higher negative expression of its respective miRNAs. Similarly, binding protein-9 (*TGA9*) from the salicylic acid pathway exhibited higher expression from 12 to 72 HAE than at the time of cutting excision (0 HAE), corresponding to the lower expression of their respective miR398b. These genes may play a significant role in the induction and initiation phase of etiolation-induced IBA-dependent AR formation. Our results are consistent with the previous findings on auxin-induced AR formation in sugarcane [87].

### 4.4. miRNAs and Their Target Genes in Sucrose Metabolic Pathways during AR Formation

In this investigation, a number of miRNAs, such as miR389a-3p, novel-m0068-5p, novel-m0650-3p, novel-m0560-3p, and miR477-3p, were discovered; these miRNAs could potentially target starch and sucrose metabolic pathways. Starch synthase is one of the enzyme classes responsible for starch biosynthesis. *SS3* is required for starch synthesis in *Arabidopsis* and *Eucalyptus globulus* AR development [88,89]. For instance, vacuolar invertase-1 and starch synthase-3 were the potential targets of miR389a-3p and novel-m0068-5p. In contrast, probable starch synthase-3 was the potential target of novel-m0560-3p, upregulated at 12 and 72 HAE; however, it was downregulated at 36 and 48 HAE. The expression of these differentially regulated genes during dark-pretreated IBA-dependent AR formation indicate that the regulation of carbohydrate metabolism and energy supply, and the regulation of sink strength, is critical for AR formation in tetraploid *R. pseudoacacia* L., as has been shown for cuttings of *Petunia*, carnation, and *Eucalyptus* [90,91,92,93]. These genes were significantly expressed during dark-pretreated etiolation-induced AR formation, and are therefore responsible for increased starch biosynthesis, and ultimately supply energy; they may also be related to sink strength for AR formation [88,89]. We observed that dark treatment significantly enhances the vacuolar invertase-1 gene compared to controls during the induction phase of AR development, after subsequent exposure of the micro-shoot cuttings to light [91]. These findings are consistent with the previous research on the induction of AR formation in apples, where the soluble sugar levels play a crucial role [94]. However, the activity of cell wall invertase-4 was the potential target of the miR477-3p gene, and was downregulated during the induction phase. Therefore, we can hypothesize that both *CWINV4* and *VI1* exhibited biphasic responses during the induction phase of tetraploid *R. pseudoacacia* L. AR development [95]. These potential genes and miRNAs may play a critical role during AR formation.

## 5. Conclusions

The present investigation revealed the importance of the integrated transcriptome, microRNA, and anatomical analysis for exploring the molecular factors and processes controlling IBA-dependent AR formation in dark-pretreated tetraploid *R. pseudoacacia* L. micro-shoot cuttings under in vitro conditions. Histological examination confirmed different stages of AR formation from 12 HAE to 72 HAE. Our findings highlighted the role of critical microRNAs and their targeted genes during AR formation at specific time points. A total of 674 known and 1099 novel miRNAs were predicted across all comparison libraries. The results further unveiled enhanced expression of 81, 162, 153, 154, 41, 9, and 77 miRNAs, and decreased expression of 67, 98, 84, 116, 19, 16, and 93 miRNAs, in different comparison groups. Overall, the data indicate that the expression of miRNAs negatively regulates the expression of targeted genes, including *GH3.9*, *SAUR70*, *ARF3*, *SCL5*, *TGA9*, *VI1*, *SS3*, *SUS3*, *SPS4F*, *CWINV4*, and *TPS5*. These findings suggest that the miRNAs control the regulation of phytohormone signal transduction, and starch and sucrose metabolism; therefore playing a critical role during AR formation in tetraploid *R. pseudoacacia* L. cuttings. Further study the molecular regulation of AR formation in dark-pretreated micro-shoot cuttings in tetraploid *R. pseudoacacia* L. would also benefit studies on other plant species. The functional analysis of the differentially regulated genes and microRNAs in AR development, and the involvement of post-transcriptional or post-translational control, should be the focus in future studies.

## Data Availability

The data are present in the manuscript and Supplementary Materials and if needed, further data will be available from the corresponding author upon request.

## Supplementary Materials

The following supporting information can be downloaded at: https://www.mdpi.com/article/10.3390/genes13030441/s1, Figure S1: Correlation analyses between miRNA-seq during the initial stage of dark pretreated IBA-dependent AR formation in tetraploid *R. pseudoacacia* micro-shoot cuttings; Figure S2: MiRNAs obtained from seven constructed libraries of dark pretreated and IBA treated micro-shoot cuttings of tetraploid *R. pseudoacacia*; Figure S3: Heatmap showing the expression pattern of differentially expressed genes (DEGs) in 15 mRNA libraries during the dark pretreated IBA-dependent AR formation in tetraploid *R. pseudoacacia* micro-shoot cuttings; Figure S4: KEGG classification of differentially expressed mRNA during dark pretreated AR formation; Figure S5: Differentially expressed mRNAs in HAE0-vs-HAE12, HAE0-vs-HAE36, HAE0-vs-HAE48 and HAE0-vs-HAE72 comparison libraries; Figures S6 and S7: The display of top 20 KEGG enriched pathway terms during dark pretreated during AR formation; Table S1: Total statistics in 15 sequenced libraries during dark pretreated IBA-dependent AR formation in micro-shoot cuttings in tetraploid *R. pseudoacacia*; Table S2: Summary of the miRNA sequence analyses; Table S3: Identification of known and novel miRNAs; Table S4: Total DE miRNAs and their target genes in plant hormones and starch and sucrose metabolism pathways during dark pretreated IBA-dependent AR formation in tetraploid *R. pseudoacacia* L.; Table S5: Total number of DE miRNAs in all seven comparison group libraries during dark pretreated IBA-dependent AR formation in tetraploid *R. pseudoacacia* L.; Table S6: Primers used for qRT-PCR assay.
