# Supplementary material for: Transcriptome Profiling Reveals Role of MicroRNAs and Their Targeted Genes during Adventitious Root Formation in Dark-Pretreated Micro-Shoot Cuttings of Tetraploid Robinia pseudoacacia L
Source: Genes (Basel). 2022 Feb 27;13(3):441. doi: 10.3390/genes13030441 (PMC8950900; doi:10.3390/genes13030441)
Supplement: Supplementary file 1 [file genes-13-00441-s001.zip › Supplementary figures 1-7.pdf]

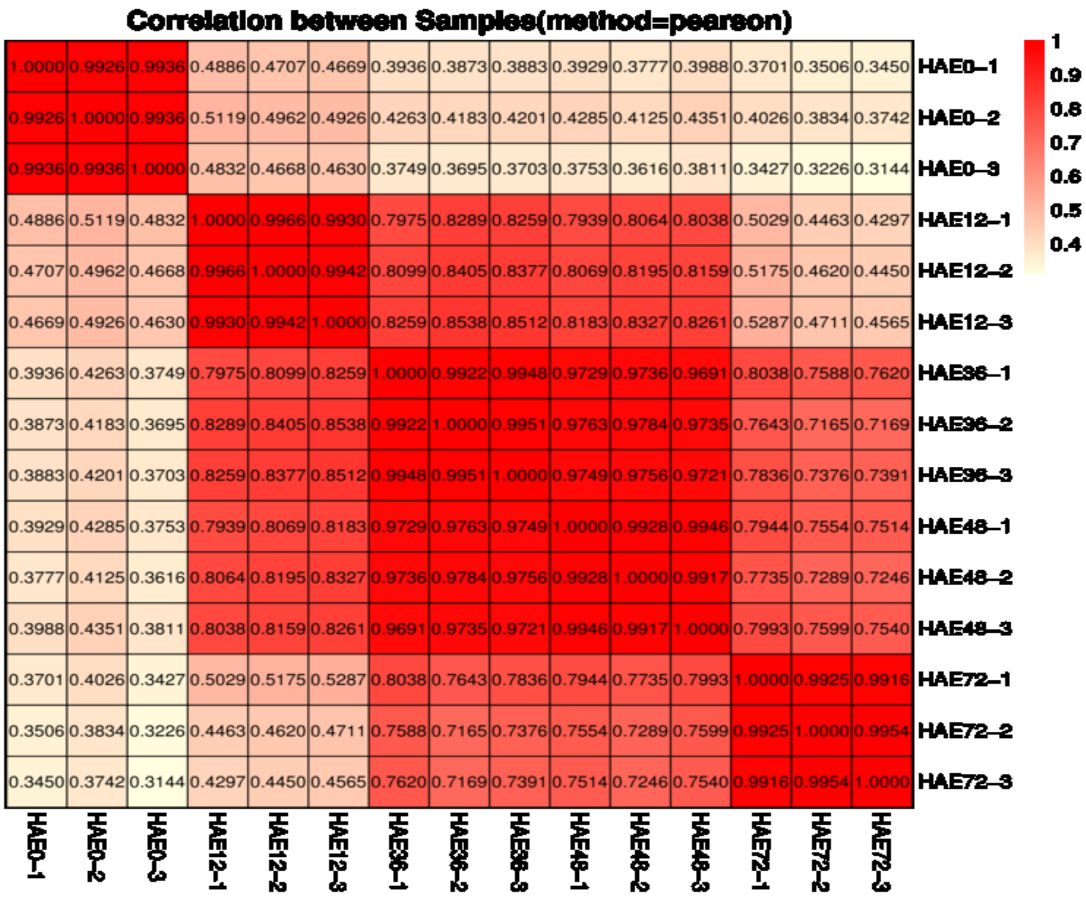

Figure S1. Correlation analysis between miRNA-seq during the initial stage of dark pretreated IBA-dependent AR formation in tetraploid *R. pseudoacacia* micro-shoot cuttings.

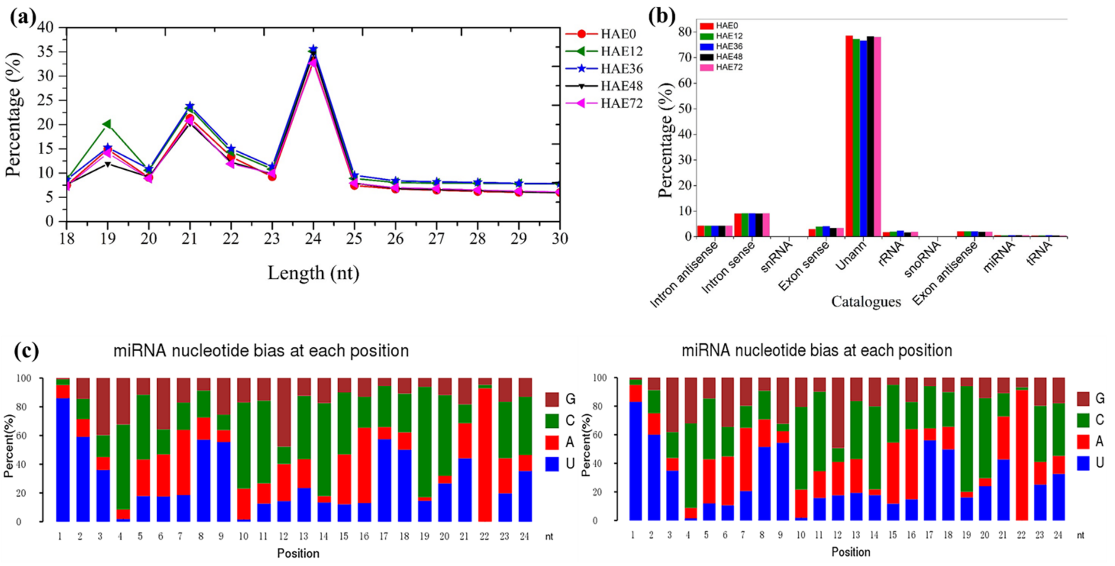

Figure S2. MicroRNAs obtained from seven constructed libraries of dark pretreated and IBA-treated (except of 0 HAE) micro-shoot cuttings of tetraploid *R. pseudoacacia* L. using high throughput sequencing (a) Length distribution of miRNAs at HAE 0, HAE 12, HAE 36, HAE 48, and HAE 72 in constructed libraries (b) Distribution of microRNA among different categories in HAE 0, HAE 12, HAE 36, HAE 48 and HAE 72 in constructed libraries (c) Nucleotide bias at each position of known and novel microRNA candidates at HAE 0, HAE 12, HAE 36, HAE 48, and HAE 72 in constructed libraries.

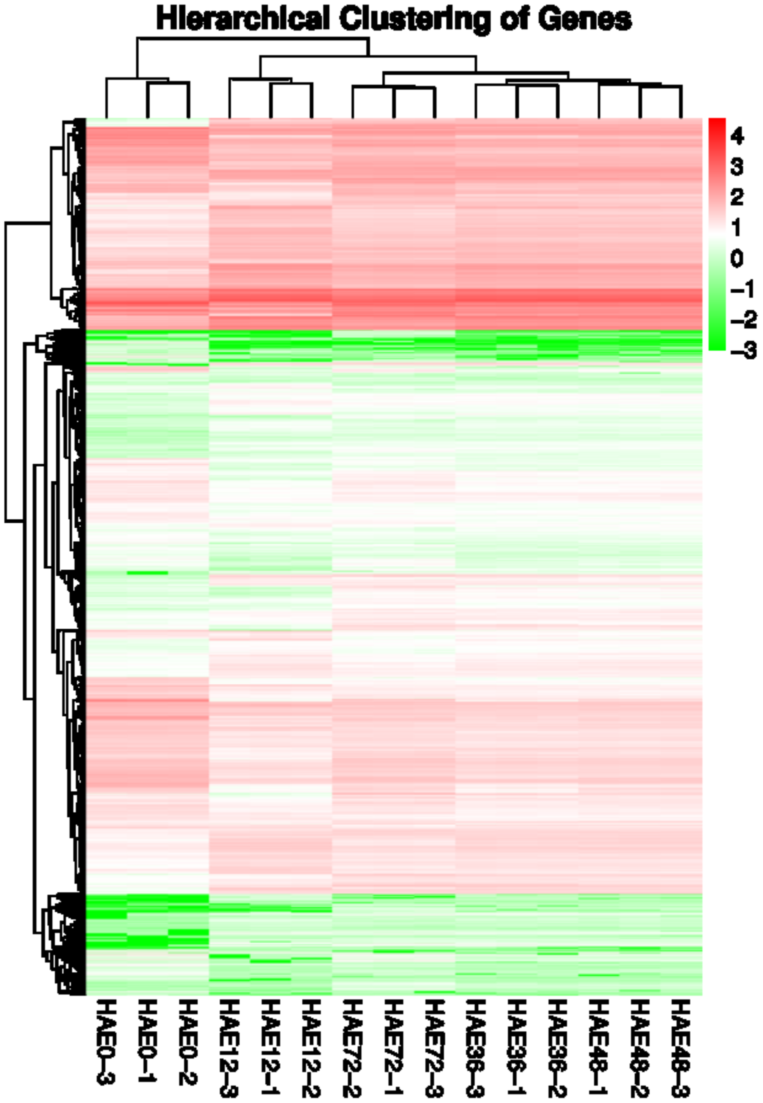

Figure S3. Heatmap showing the expression pattern of differentially expressed genes (DEGs) in 15 mRNA libraries during the dark pretreated IBA-dependent AR formation in tetraploid *R. pseudoacacia* micro-shoot cuttings.

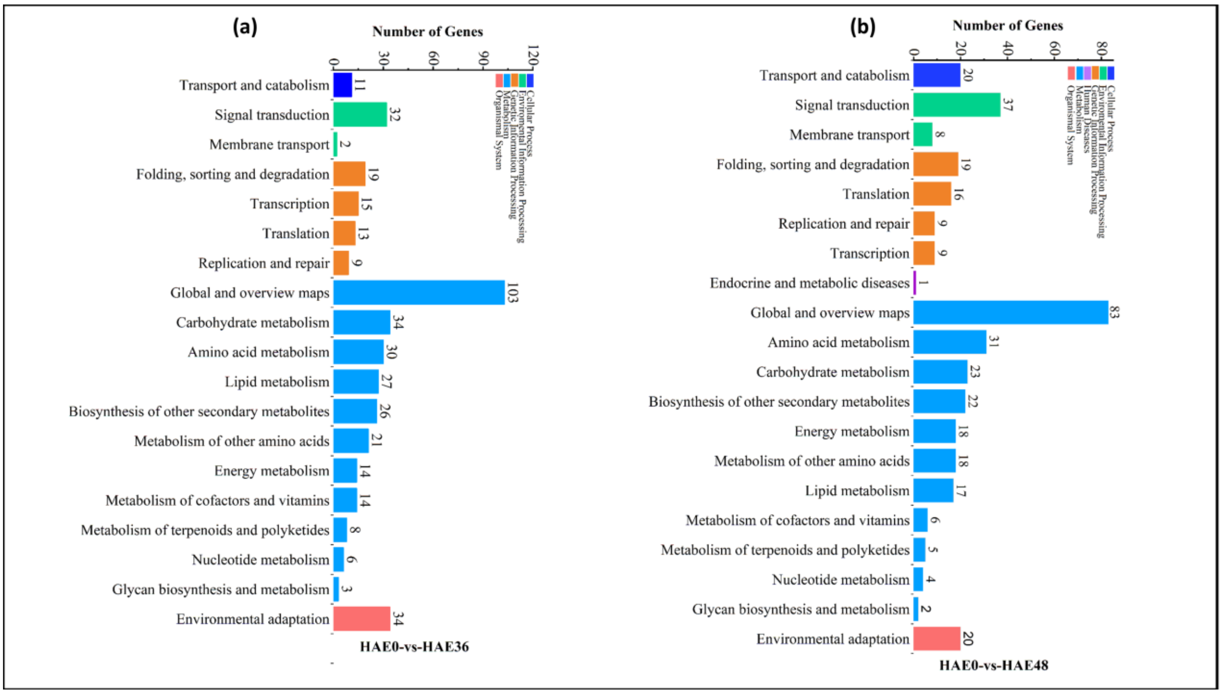

Figure S4. KEGG classification of differentially expressed mRNAs during dark pretreated IBA-dependent AR root formation in tetraploid *R. pseudoacacia* micro-shoot cuttings. The X axis showed the number of differentially expressed mRNAs, and the Y axis showed the second KEGG pathway terms. The pathways terms were indicated using different colors, and were grouped together on the X axis on the left side **(a)** The number of differentially expressed mRNAs in HAE0-vs-HAE36 comparison group libraries **(b)** The number of differentially expressed miRNAs in HAE0-vs-HAE48 comparison group libraries.

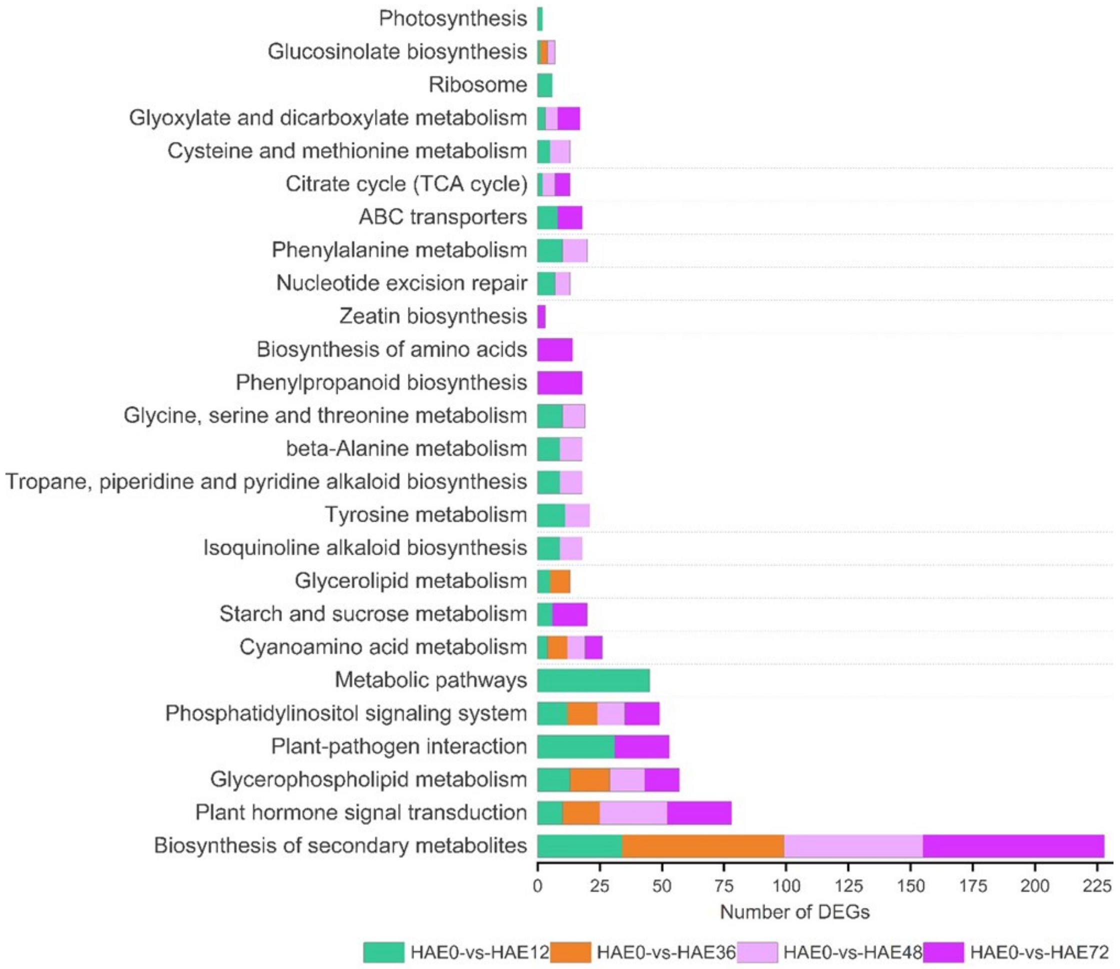

Figure S5. Differentially expressed mRNAs in HAE0-vs-HAE12, HAE0-vs-HAE36, HAE0-vs-HAE48, and HAE0-vs-HAE72 comparison libraries. The enrichment of DEGs in the top 20 KEGG pathways. The light green, orange, pink, and magenta colors indicate the number of DEGs.

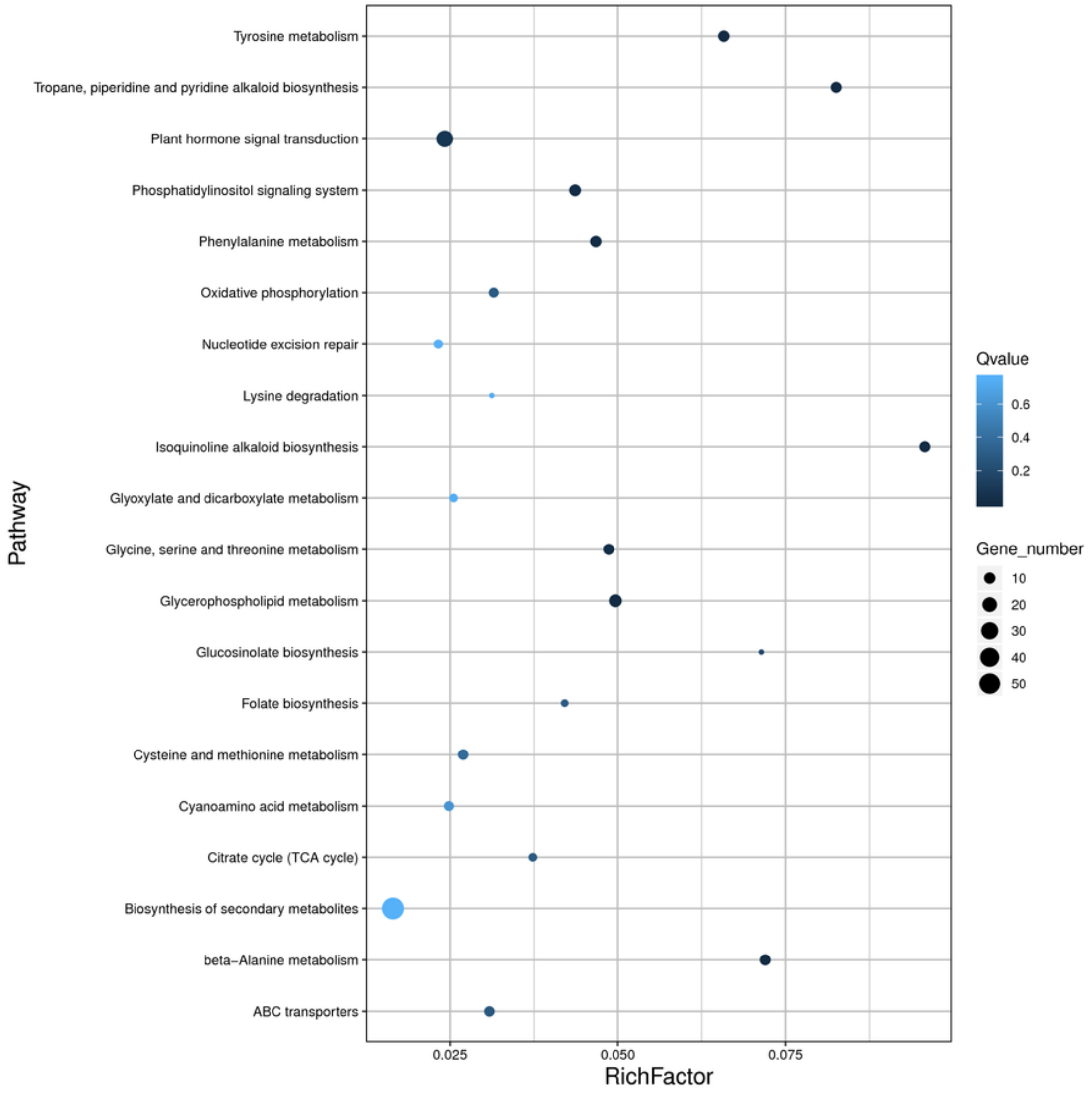

Figure S6. The display of the top 20 KEGG enriched pathway terms during dark pretreated IBA-dependent AR root formation in tetraploid *R. pseudoacacia* micro-shoot cuttings. The rich factor was the ratio of differentially expressed miRNAs numbers annotated in this pathway term to all gene numbers annotated in this pathway term, and the greater the rich factor, the greater the degree of enrichment. The display of the top 20 enriched pathway terms in HAE0-vs-HAE48 comparison group libraries.

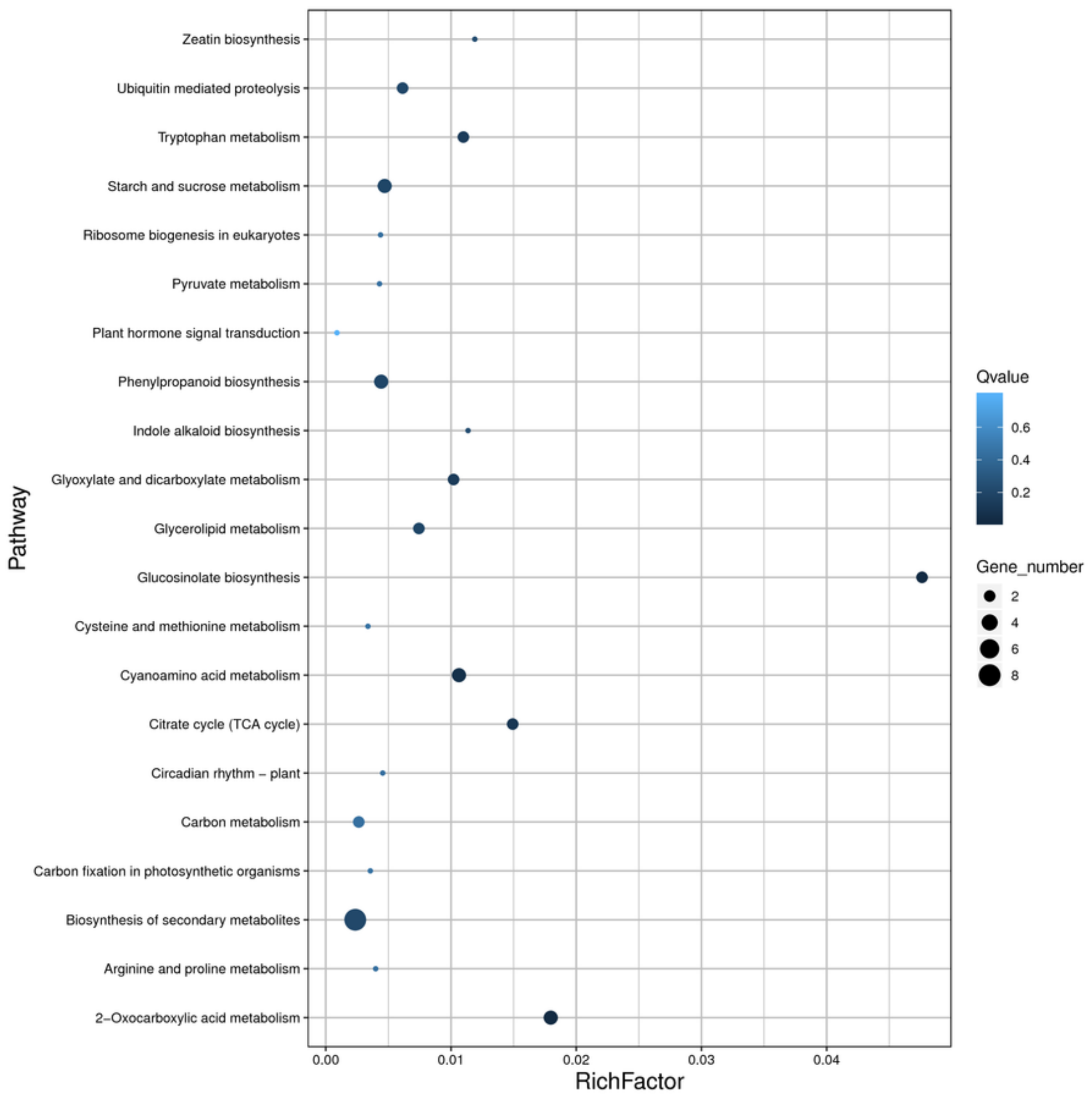

Figure S7. The display of the top 20 KEGG enriched pathway terms during dark pretreated IBA-dependent AR root formation in tetraploid *R. pseudoacacia* micro-shoot cuttings. The rich factor was the ratio of differentially expressed miRNAs numbers annotated in this pathway term to all gene numbers annotated in this pathway term, and the greater the rich factor, the greater the degree of enrichment. The display of the top 20 enriched pathway terms in HAE36-vs-HAE48 comparison group libraries.
